# Pre-earthquake anomaly extraction from borehole strain data based on machine learning

**DOI:** 10.1038/s41598-023-47387-z

**Published:** 2023-11-16

**Authors:** Chengquan Chi, Chenyang Li, Ying Han, Zining Yu, Xiang Li, Dewang Zhang

**Affiliations:** 1 School of Information Science and Technology, Hainan Normal University, Haikou, China; 2 College of Geography and Environmental Science, Hainan Normal University, Haikou, China; 3 College of Information Science and Engineering, Ocean University of China, Qingdao, China

**Keywords:** Natural hazards, Solid Earth sciences

## Abstract

Borehole strain monitoring plays a critical role in earthquake precursor research. With the accumulation of observation data, traditional data processing methods struggle to handle the challenges of big data. This study proposes a segmented variational mode decomposition method and a GRU-LUBE deep learning network based on machine learning theory. The algorithm enhances data correlation during decomposition and effectively predicts borehole strain data changes. We extract pre-earthquake anomalies from four-component borehole strain data of the Guza station for two major earthquakes in Sichuan (Wenchuan and Lushan earthquakes), obtaining more comprehensive anomalies than previous studies. Statistical analysis reveals similar abnormal phenomena in the Guza station’s borehole strain data before both earthquakes, suggesting shared crustal stress accumulation and release patterns. These findings highlight the need for further research to improve earthquake prediction and preparedness through understanding underlying mechanisms.

## Introduction

Large earthquakes are driven by continuously changing tectonic stresses, which occur after a long-term preparation phase composed of different stages of seismic activity evolution^1^. Considerable work remains to be done in the study of earthquake precursors. Pre-earthquake anomaly extraction is a prerequisite for earthquake precursor analysis. Over the past few decades, a significant volume of earthquake precursor observation data has been accumulated worldwide, thus providing a solid data foundation for earthquake precursor research.

Investigating precursory data of earthquakes holds significant importance in enhancing our understanding of seismic activities and their potential consequences. By meticulously analyzing patterns and trends, researchers can identify early warning signs that may offer valuable insights into impending seismic events. Moreover, the continuous advancements in data analysis techniques contribute to refining predictive models, fostering collaboration among scientists, and ultimately improving global seismic hazard assessment and management. Worldwide, many scholars have utilized various earthquake precursor data to analyze specific earthquake cases in academic research. Wang et al. compared theoretical and observed GPS values to identify the geodetic anomaly pre-ceding the Lushan earthquake and verified that the pre-earthquake anomalies in GPS data were related to the Lushan earthquake^2^. De Santis et al. analyzed electron density and magnetic field data measured over 4.7 years by the Swarm three-satellite constellation to identify possible in-situ ionospheric pre-earthquake anomalies of large earthquakes from different angles, and they determined that in-situ ionospheric anomalies were correlated with earthquakes^3^. Skelton et al. analyzed the stable isotope ratios and dissolved element concentrations of groundwater obtained from a borehole in northern Iceland between 2008 and 2013 and found that changes in groundwater chemistry were associated with earthquakes^4^. Hattori et al. used principal component analysis to analyze the pre-earthquake anomalies in ULF geomagnetic data, and the results showed that these features are likely to be correlated with large earthquakes^5–8^. Fan et al. used a nonnegative tensor decomposition (NTD) approach to analyze the Swarm Satellite magnetic field data for the 2015 Mw 7.8 Nepal Earthquake and had extracted anomalous phenomena related to earthquakes^9^. Additionally, research has been conducted on other areas like groundwater^10,11^, gravity^12^, ozone anomaly indices^13^, and outgoing long-wave radiation (OLR) data^14^, all of which have produced significant findings.

Since the “Plate Boundary Observatory” (PBO) was launched in the United States, borehole strain observations have received unprecedented attention because of their high resolution and high sensitivity^15–17^. In ground monitoring systems, crustal deformation observations rank among the most crucial precursor observation items. Borehole strain observations represent an important method of studying crustal deformation and changes in the in-situ stress field. Crustal deformations can be observed under the action of a regional stress field. Installed deep within bedrock, borehole strainmeters can record both continuous stress and strain measurements, establishing themselves as essential tools for monitoring crustal deformation. High-resolution records provided by borehole strainmeters allow for the detection of subtle strain variations.

Borehole strain observations can record the strain changes related to earthquakes, thus providing a possible method of extracting strain anomalies before earthquakes. However, borehole strain observations are susceptible to interference from the external environment because of their high-accuracy and wide-band characteristics. Researchers have studied the removal of external interference from borehole strain data. Ren et al. studied the influence of water level fluctuations of the Dadu River on a Guza borehole strainmeter and provided a basis for removing the influence of borehole strain data^18^. Chi and Yu et al. used PCA^19^, state space equation^20^ and VMD^21^ methods to remove the strain response due to air pressure, solid tides and changes in water level to preferentially isolate non-tectonic disturbances. Qiu et al. used high-pass filtration and overrun rate analyses to remove the long-period signal and extracted borehole strain anomalies before the large earthquakes^20^.

On the basis of removing interference, scholars have conducted pre-earthquake anomaly analyses of borehole strain data for different earthquakes. Shi et al. observed the variations of co-seismic static stress deviations and showed for the first time that they are consistent with theoretical predictions by observing borehole strain, which is of great significance for earthquake prediction^22^. Gong et al. studied borehole strain data and analyzed the seismogenic structure of the Hutubi earthquake in detail^23^. Chi et al. used VMD and PCA to detect the pre-earthquake anomaly of the Wenchuan earthquake and verified the correlation between the earthquakes and anomalies^24^. Zhu et al. detected the pre-earthquake anomaly of the Lushan earthquake by calculating eigenvalues and eigenvectors^25^.

As the accumulation and complexity of earthquake precursor observation data continue to grow, traditional signal processing techniques are becoming overwhelmed. Conventional filters, due to variations in data frequency and dynamic shifts, often omit or blur critical information. The VMD technique, employed for data decomposition, frequently encounters memory overflow issues with large datasets. This paper introduces the SVMD method, designed to circumvent memory limitations while preserving data correlations.

In recent years, machine learning techniques have emerged as promising tools for studying earthquake precursory data. Current research leverages algorithms like support vector machines^26–28^, neural networks^29–38^, a random forest (RF) model^39,40^ and decision trees^41^ have shown potential in enhancing seismic event prediction, such as estimating time, location, and magnitude, while also reducing false alarms. While significant progress has been made, challenges remain in terms of data quality, scarcity and heterogeneity. The ongoing development and refinement of machine learning models, alongside the increasing availability of high-quality data, aim to improve the accuracy and reliability of earthquake forecasting.

**Figure 1:**
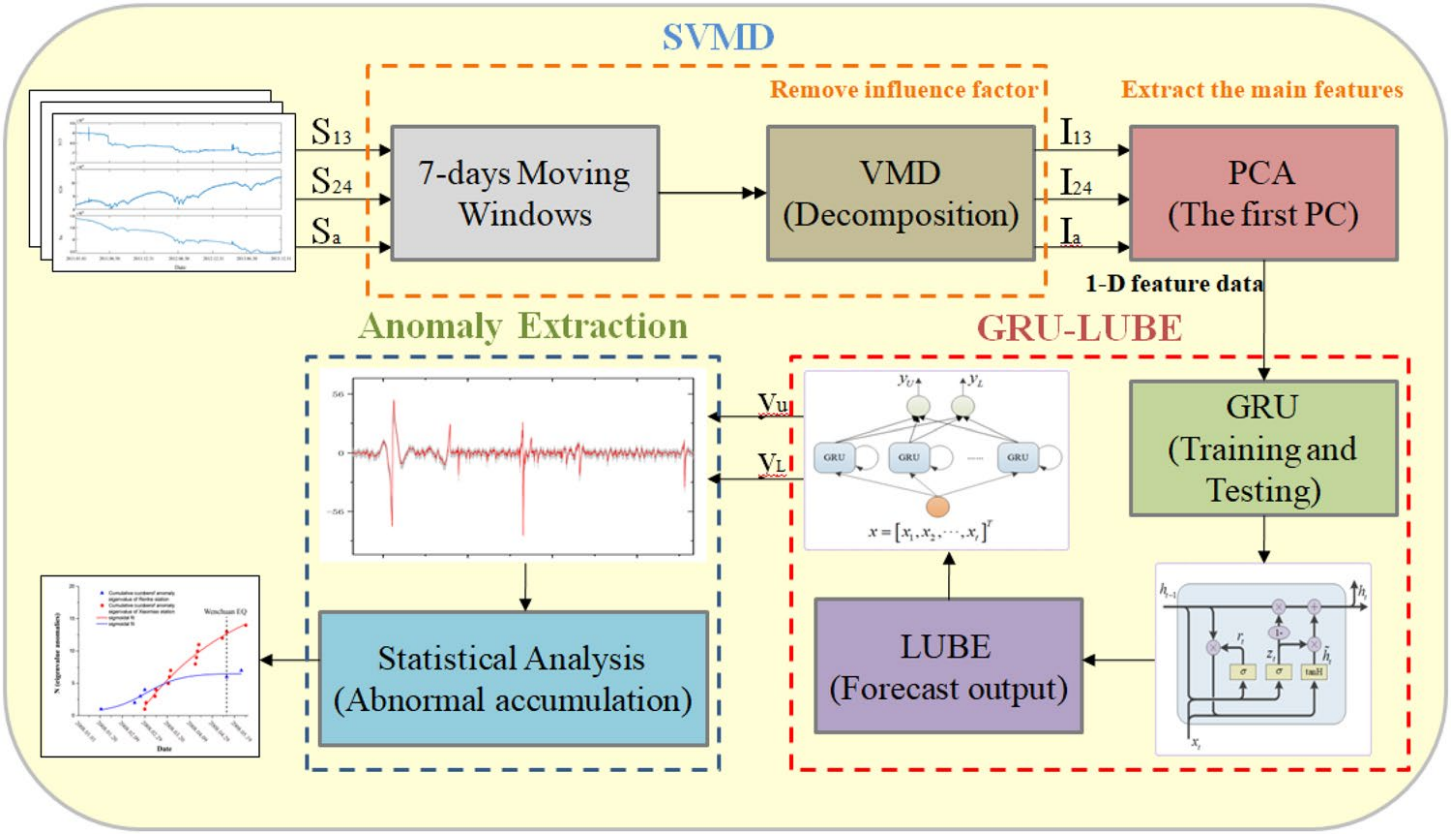
Structure of borehole strain data processing and anomalies extraction.

In this paper, a GRU-LUBE network was proposed to extract pre-earthquake anomalies from borehole strain data and this article takes the Wenchuan earthquake and Lushan earthquake as examples to analyze the data of Guza station and compare the analysis results of other two stations to determine the effectiveness of the algorithm. The GRU-LUBE network utilized in this study is not only adept at capturing the characteristics of the borehole strain data but also excels in constructing prediction intervals by calculating the upper and lower bounds of the forecasted data, enabling efficient anomaly detection. Through this study, more accurate pre-earthquake anomaly information can be provided for earthquake warning systems, improving the accuracy and timeliness of earthquake prediction. The flow of this paper is shown in Fig. 1.

As is shown in Fig. 1, first of all, Segmented Variational Mode Decomposition (SVMD) is used to decompose the borehole strain data to remove the influencing factors (include annual trends, solid tidal responses, atmospheric, pressure, rainfall, etc.). Second, a gated recurrent unit (GRU) network is constructed to forecast the borehole strain data and the Upper and Lower Bound Estimation (LUBE) algorithm is used to compare the real data with the predicted data to realize data anomaly extraction. Finally, statistical methods and comparative analysis of multiple stations are used to analyze and identify anomalies before earthquakes.

## Methods

### Segmented variational mode decomposition (SVMD)

Variational mode decomposition (VMD) is based on solving the variational problems of classical Wiener filtering and Hilbert transform, it can adaptively decompose signals into several finite-bandwidth intrinsic mode functions by iteratively searching for the optimal solution of the variational model^42^. VMD is adaptive to the processing of nonlinear and nonstationary signals, it transforms the data decomposition problem into a variational problem. The resulting constrained variational problem is as follows:

1 $$ \min_{{\{ u_{k} \} ,\{ \omega_{k} \} }} \left\{ {\sum\limits_{k} {\left\| {\alpha_{t} \left[ {\left( {\delta (t) + \frac{j}{\pi t}} \right) * u_{k} (t)} \right]e^{{ - j\omega_{k} t}} } \right\|_{2}^{2} } } \right\}\quad s.t.\sum\nolimits_{k} {u_{k} } = f $$

In Eq. (1), ${u}_{k}$ is the *k*-th intrinsic mode function (or mode). It represents one of the decomposed components of the original signal, which is a band-limited oscillatory function centered around a specific frequency; ${\omega }_{k}$ is the center frequency associated with the *k*-th mode ${u}_{k}$, it describes the central oscillation frequency of the corresponding mode; where $\delta (t)$ is the Dirac distribution. The modes *u*_*k*_ and their corresponding center frequency $\omega_{k}$ can be updated as follows:

2 $$ u_{k}^{n + 1} \leftarrow \arg \;\min L_{{\omega_{k} }} (u_{i < k}^{n + 1} ,\;u_{i \ge k}^{n + 1} ,\;\omega_{i}^{n} ,\;\lambda^{n} ) $$

3 $$ \omega_{k}^{n + 1} \leftarrow \arg \;\min L_{{\omega_{k} }} (u_{i}^{n + 1} ,\;u_{i < k}^{n + 1} ,\;\omega_{i \ge k}^{n} ,\;\lambda^{n} ) $$

However, the VMD method conducts a global search and solves variational problems, which may cause computational challenges, such as slow processing speed and computer memory limitations, due to the large amount of data involved. Conventional data segmentation approaches can be employed, but they may result in the loss of data correlation between segments. To address these issues, we propose a Segmented Variational Mode Decomposition (SVMD) method. The underlying principle of the SVMD method is illustrated in Fig. 1, offering a solution that maintains data correlation during segmentation while effectively handling large datasets.

**Figure 2:**
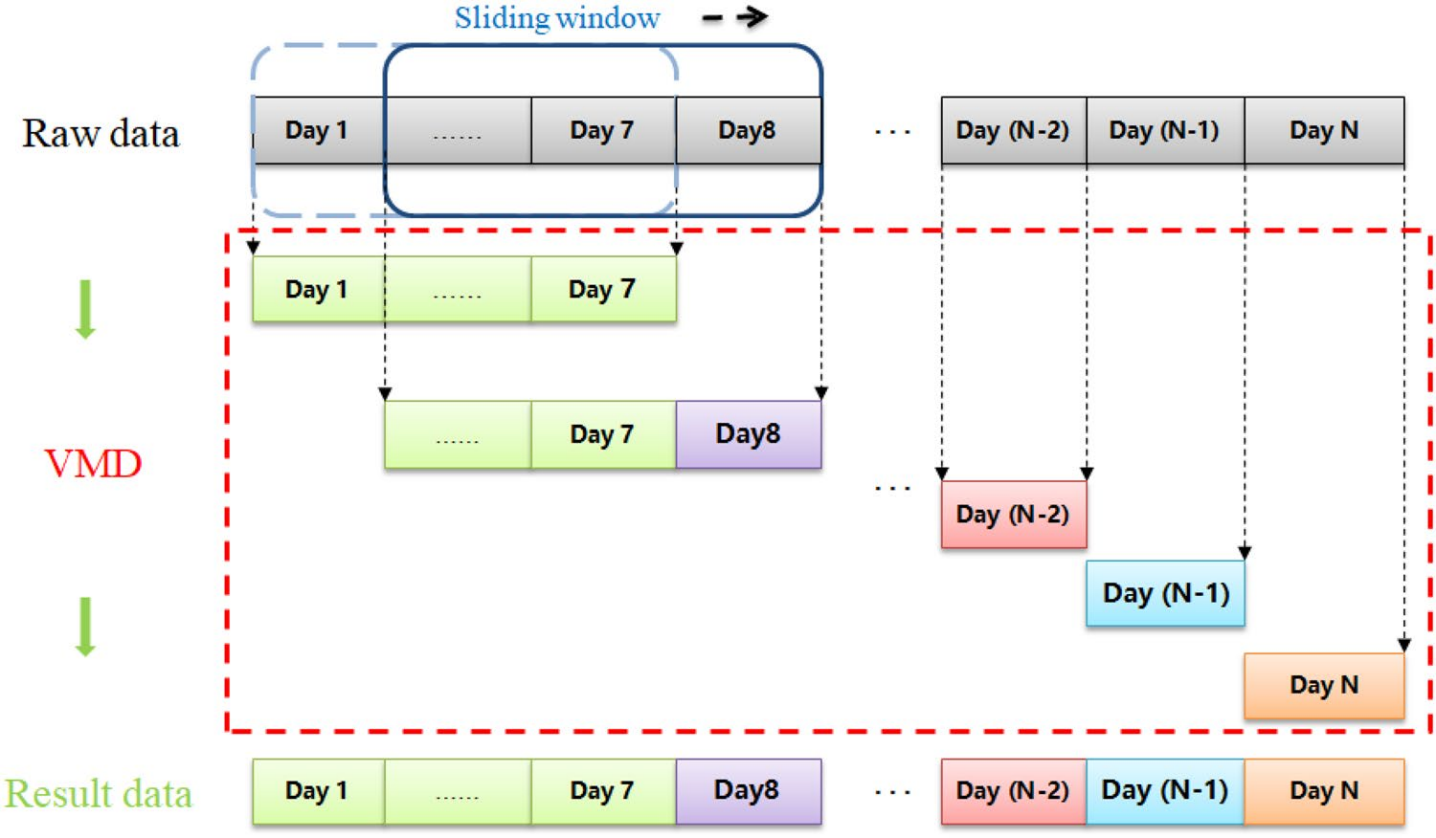
Segmented variational mode decomposition principal diagram.

As shown in Fig. 2, we implemented data segmentation by adding a sliding window with a window length of 7 days and a sliding step of one day. All data from the first de-composition were retained and only the decomposition results of the last day were retained for the remainder of the decomposition process. This method not only retains the correlation between data but also greatly reduces the time consumed by data decomposition, preventing the problem of algorithms from failing to run owing to large amounts of data.

### GRU-LUBE network

In recent years, the application of advanced machine learning techniques has significantly improved our understanding and prediction of natural disasters, including earthquakes.

The activation function of $\sigma ( \cdot )$ is a sigmoid function. The output of the reset gate and current input pass through the activation function (tanh) to obtain the candidate hidden state. The update gate is used to determine the degree of retention of the current output state relative to the previous state. The formulae used are as follows:

4 $$ r_{t} = \sigma (\omega_{r} \cdot [h_{t - 1} ,\;x_{t} ]) $$

5 $$ \tilde{h}_{t} = \tanh (\omega \cdot [r_{t} * h_{t - 1} ,\;x_{t} ]) $$

6 $$ z_{t} = \sigma (\omega_{z} \cdot [h_{t - 1} ,\;x_{t} ]) $$

where $h_{t - 1}$ is the hidden state at the last moment, $x_{t}$ is the network input, $r_{t}$ is the output of the reset gate, $\tilde{h}_{t}$ is the candidate hidden state and $z_{t}$ is the output of the update gate.

The Lower and Upper Bound Estimation (LUBE) method is a nonparametric method that directly constructs prediction intervals and offers significant advantages in extracting anomalies from earthquake precursor data. LUBE is a statistical approach used for anomaly detection and outlier analysis by calculating the lower and upper bounds within a dataset, thereby identifying anomalous points. Accurate detection of anomalies in earthquake precursor data is crucial for improving the precision of earthquake early warning systems. In this paper, the GRU neural network is applied to the LUBE method and the two outputs correspond to the upper and lower bounds of the interval^43^.

The mode of the GRU-LUBE is shown in Fig. 1, where $y_{U}$ and $y_{L}$ are the upper and lower bounds of the model output, respectively. The model output can be expressed as follows:

7 $$ \left[ {\begin{array}{*{20}c} {y_{U} } \\ {y_{L} } \\ \end{array} } \right] = \left[ {\begin{array}{*{20}c} {\sum\limits_{j = 1}^{m} {w_{ho}^{j1} \cdot g\left( {\sum\limits_{i - 1}^{n} {x_{i} w_{in}^{ij} + b^{j} } } \right) + \sum\limits_{i = 1}^{n} {x_{i} w_{io}^{i1} } } } \\ {\sum\limits_{j = 1}^{m} {w_{ho}^{j2} \cdot g\left( {\sum\limits_{i - 1}^{n} {x_{i} w_{in}^{ij} + b^{j} } } \right) + \sum\limits_{i = 1}^{n} {x_{i} w_{io}^{i2} } } } \\ \end{array} } \right] $$

where $x_{i}$ is the input vector; $w_{ih}$ is the weight between the input layer neurons and the hidden layer neurons, which is an $n \times m$ matrix; b is the threshold of the hidden layer neurons; $w_{ho}$ is the weight between the hidden layer neurons and the output layer neurons, which is an $m \times 2$ matrix; and $w_{io}$ is the weight between the input layer neurons and the output layer neurons, which is an $n \times 2$ matrix.

In this paper, the input to the GRU-LUBE network is the pre-processed borehole strain data and the output is the predicted upper and lower threshold values. Data from relatively stable time periods are used as training data, with a data length of at least 6 months.

The execution speed of the algorithm proposed in this paper is primarily influenced by the SVMD decomposition phase and the training phase of the GRU network. It's recommended that, with the advancement of computer hardware as a prerequisite, the time consumption caused by the SVMD decomposition and the data training of the GRU network should be addressed using a distributed computing framework to distribute the computational load. Additionally, performing calculations on GPUs will further enhance the processing speed.

## Observation data and earthquakes

### Observation data

Borehole strainmeters are designed to place sensors within boreholes to monitor deformation. In comparison to the Earth’s size, the deformation observed in the crust represents a minute portion, which can be approximated as the deformation observation result of a singular point. Four-component borehole strain observations represent a type of relative observation, capable of detecting changes in the target observation but not providing a complete measurement of the target observation. This characteristic is determined by the underlying principle of its model^44^.

With one additional measurement, a simple relationship between the four measurements can be obtained using Eq. (8):

8 $$ S_{1} + S_{3} = k(S_{2} + S_{4} ) $$

which is the self-consistency equation of the YRY-4 borehole strainmeter. This equation can be used to estimate the credibility of data. k is the self-consistent coefficient and k = 1 under ideal circumstances. We believe that the data are reliable when k ≥ 0.95.

Only three independent variables are considered under plain strain conditions at or near the Earth’s surface. Therefore, we can derive various strains from the Guza recordings. The formulae used are as follows:

9 $$ \left\{ {\begin{array}{*{20}l} {S_{13} = S_{1} - S_{3} } \\ {S_{24} = S_{2} - S_{4} } \\ {S_{a} = S_{1} + S_{2} + S_{3} + S_{4} } \\ \end{array} } \right. $$

where $S_{a}$ represents the areal strain, $S_{13}$ and $S_{24}$ represent the two independent shear strains. The areal strain and shear strain are of physical significance; therefore, this study investigates areal strain and shear strain data.

### Earthquakes and stations

We looked into the Wenchuan earthquake and the Lushan earthquake for this essay. Wenchuan County, Sichuan, experienced a Ms8.0 earthquake on May 12 at 14:28 (UTC + 8). 31.01 N and 103.42 E were the coordinates of the epicenter. The focal depth was roughly 14 km, according to data released by the China Earthquake Networks Center of the China Earthquake Agency. On April 20, 2013, at 08:02, a Ms7.0 earthquake struck Lushan County in Ya'an, Sichuan. 30.277° N and 102.937° E were the coordinates of the epicenter. The Chinese Earthquake Administration's China Earthquake Networks Center reported that the earthquake's Ms7.0 magnitude and roughly 13 km focal depth.

**Figure 3:**
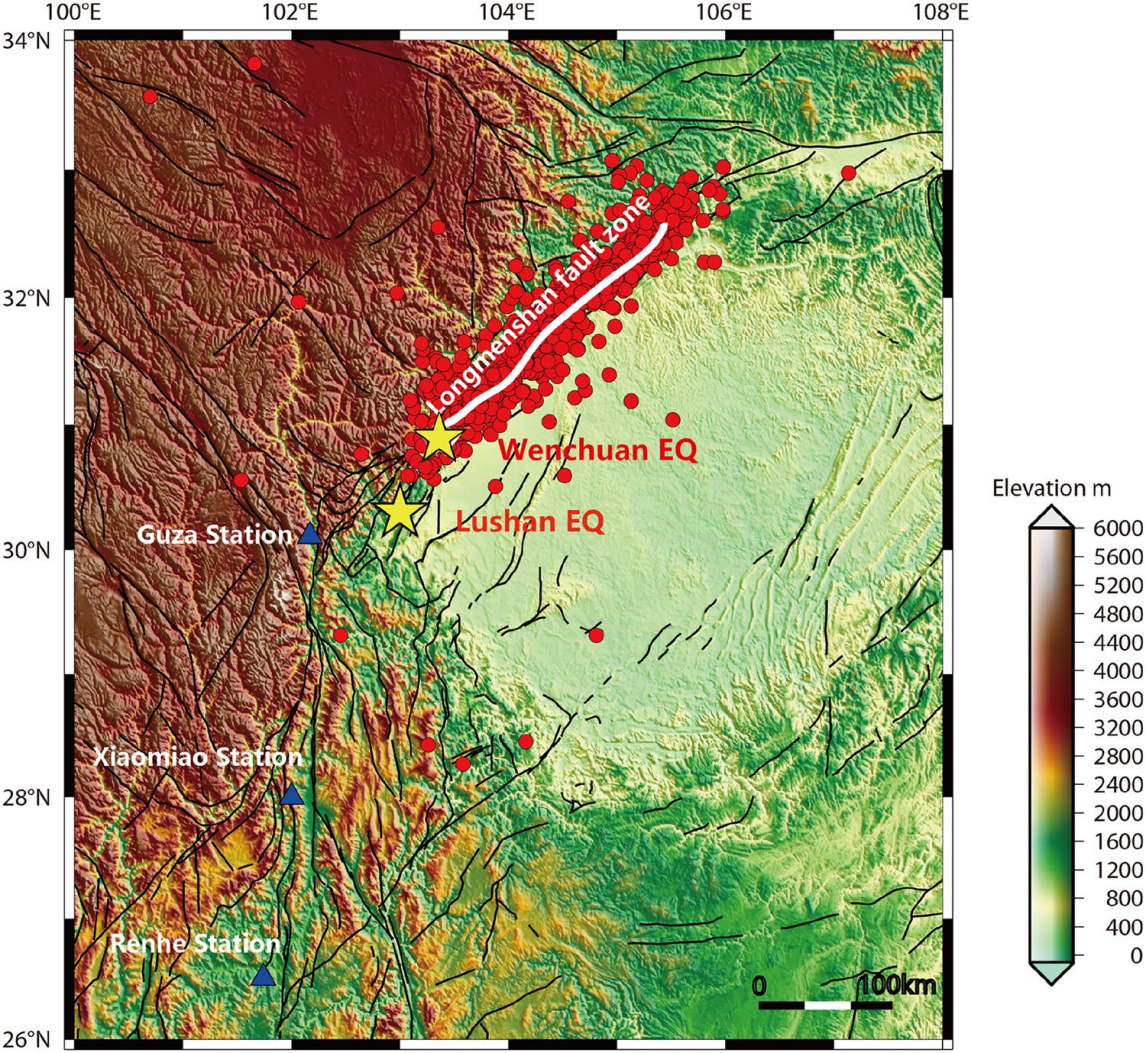
Location map showing three stations and the Wenchuan and Lushan Earthquake epicenter. The yellow pentagrams denote the epicenter locations of the two earthquakes, while the blue triangles indicate the positions of the three stations, the black curves indicate the faults. Map is generated by GMT software, v. 4.5.18 (https://gmt-china.org/).

As is shown in Fig. 3, Guza Station is the nearest to the two earthquakes, followed by Xiaomiao Station and Renhe Station. Table 1 shows the locations of stations and the rock type. The installation depth of the four-component borehole strainmeters at all three stations exceeds 40 m and they are the three closest borehole strain observation stations to the epicenters of the Wenchuan and Lushan earthquakes. The instruments at the three stations operate stably and have good data quality, the data met the self-consistent coefficient k ≥ 0.95 criterion.

**Table 1:** List of the surrounding environment of the three stations and their distances from the two earthquakes.

Station	Locations		Installation depth (m)	Distance (Wenchuan EQ) (km)	Distance (Lushan EQ) (km)	Rock type
Guza	102.18°E	30.12°N	41.00	154	73	Granodiorite
Xiaomiao	102°E	28°N	45.35	362	268	Siltstone
Renhe	101.74°E	26.51°N	61.50	526	434	Quartz diorite

## Data processing

**Figure 4:**
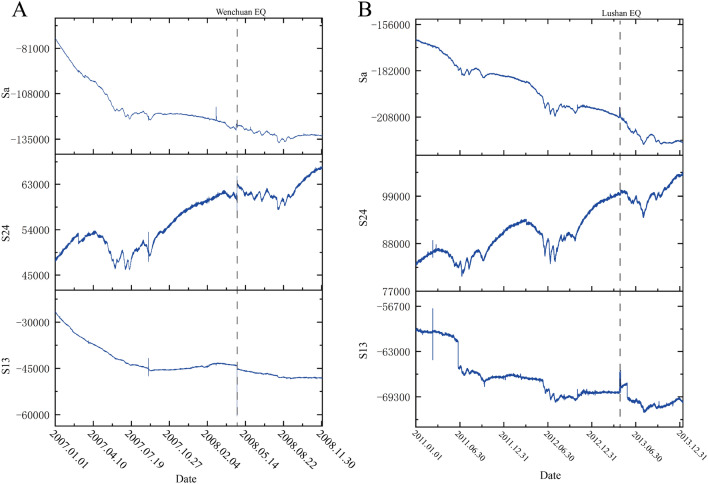
Areal strain data ($S_{a}$) and shear strain data ($S_{13}$ and $S_{24}$). (**A**) is the borehole strain data of Guza Station from 2007 to 2008, the dashed line represents the moment of the Wenchuan earthquake occurred; (**B**) is the borehole strain data of Guza Station from 2011 to 2013, and the dashed line represents the moment of the Lushan earthquake occurred.

We selected borehole strain data from Guza station from 2007 to 2008 and from 2011 to 2013 for anomaly extraction before Wenchuan earthquake and the Lushan earthquake, respectively. The data met the self-consistent coefficient k ≥ 0.95 criterion; therefore, we believe that the data are reliable. First, we converted the four-component borehole strain observational data into one areal strain ($S_{a}$) and two shear strains ($S_{13}$ and $S_{24}$). The area land shear strain data are presented in Fig. 4. As is shown in Fig. 4, the data of Guza station has obvious trend changes and annual cycle changes, except for observable co-seismic changes, no obvious pre-earthquake anomalies can be observed.

**Figure 5:**
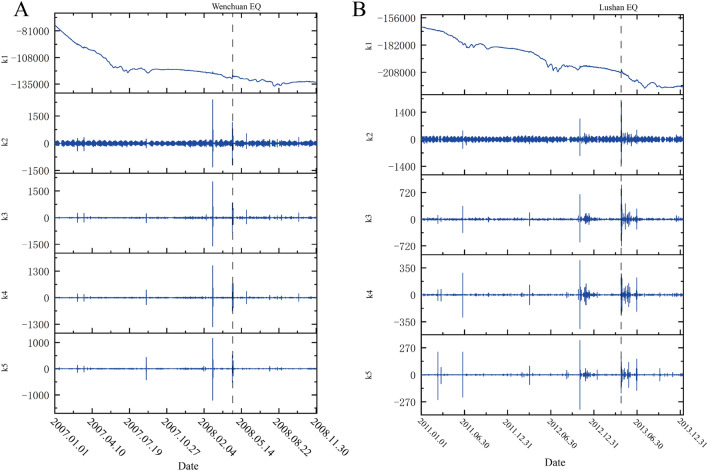
Decomposition results of $S_{a}$ by using SVMD. (**A**) is the decomposition result of borehole strain data at Guza Station in 2007 and 2008, and the dashed line represents the moment of the Wenchuan earthquake occurred; (**B**) is the decomposition result of borehole strain data at Guza Station from 2011 to 2013, and the dashed line represents the moment of the Lushan earthquake occurred.

Next, we use SVMD to decompose $S_{13}$, $S_{24}$ and $S_{a}$, respectively. The number of decomposition layers is 5, the moderate bandwidth constraint α was 2000 and the tolerance range of the convergence criterion^20^ was 10^−7^. Taking $S_{a}$ data as an example to demonstrate the decomposition results. Figure 5 shows the decomposition results for $S_{a}$.

As shown in the Fig. 5, we decomposed $S_{a}$ into five components. The first component $k_{1}$ represents the trend term and the frequency of $k_{2}$ is mainly concentrated at 1.157 × 10^−5^ Hz and 2.232 × 10^−5^ Hz, which correspond to the semidiurnal wave and diurnal wave frequencies of Earth tides, respectively. It is noticeable that the removal of Gauss noise is achieved with a Wiener filter on each mode during the decomposition progress.

We remove $k_{1}$ and $k_{2}$ components and reconstruct the remaining data to retain as much anomaly information as possible. The same treatment process was applied for $S_{13}$ and $S_{24}$, the results were similar. Short-period changes caused by crustal deformation components of $S_{13}$, $S_{24}$ and $S_{a}$ were used to extract the main features by PCA. The first principal component was calculated to represent the principal characteristics of the signals.

## Results

In this study, GRU and LUBE were used to extract pre-earthquake anomalies from borehole strain data of the Wenchuan and Lushan earthquakes. For the GRU network, the number of neurons in the hidden layer was nine, the number of neurons in the output layer was two and the number of parameters to be trained was 30 because the parameters inside the GRU were randomly generated and not updated. The two output layers correspond to the upper and lower bounds of the model output, respectively, and the confidence level was 90%, with points beyond this range considered abnormal points.

**Figure 6:**
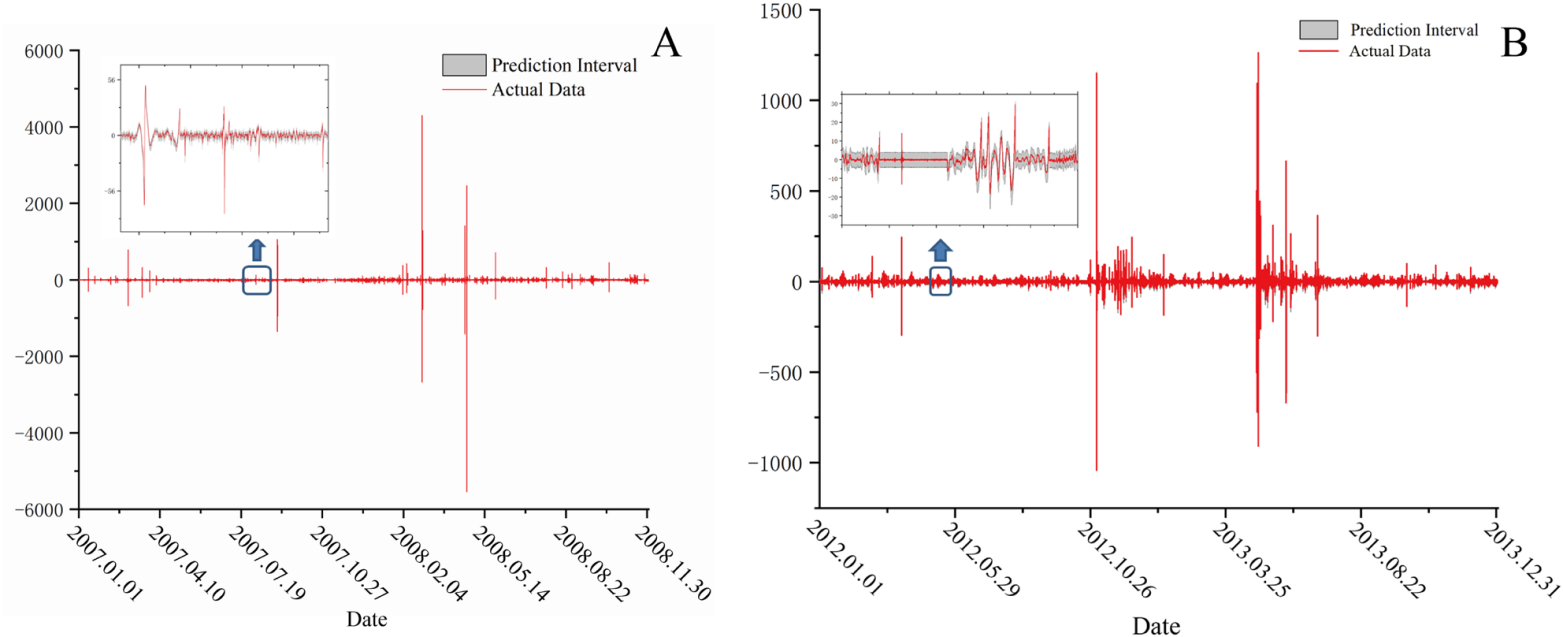
Prediction results by the GRU-LUBE network. (**A**) is the Wenchuan earthquake prediction result, The blue arrow points to the detailed section within the blue box; (**B**) is the Lushan earthquake prediction result, The blue arrow points to the detailed section within the blue box.

For Lushan earthquake, the borehole strain data from Guza in 2011 were used as training data, and borehole strain data of 2012 and 2013 were used as test data. For Wenchuan earthquake, the borehole strain data from Guza in 2011 were used as training data and borehole strain data of 2007–2008 were used as test data. Research on both earthquakes chose 2011 data as training data because of the relatively consistent borehole strain data and lack of significant earthquakes in that year. The prediction results by GRU-LUBE network is shown in Fig. 6.

As shown in Fig. 6, the red line represents the data of the actual data and the gray area represents the predicted result. As indicated by the details in the figure, the GRU-LUBE network can well predict the change form of the borehole strain data.

In the detection of abnormal borehole strain data, we consider points that exceed the upper and lower bounds of the interval as anomalies. To determine whether a particular day has anomalies, the following two conditions must be met simultaneously: (a) there must be at least 15 anomaly points within a 30-min period. (b) The difference between the center point of the upper and lower bounds interval and the actual value of the anomaly point must be greater than 1.5 times the interval width and there must be more than three such points within that 30-min period.

In order to better express the changing characteristics of abnormal days, this article calculates the cumulative number of abnormal characteristic values over time, the formula is as follows:

10 $$ N(t) = \sum {N(d(t))} $$

**Figure 7:**
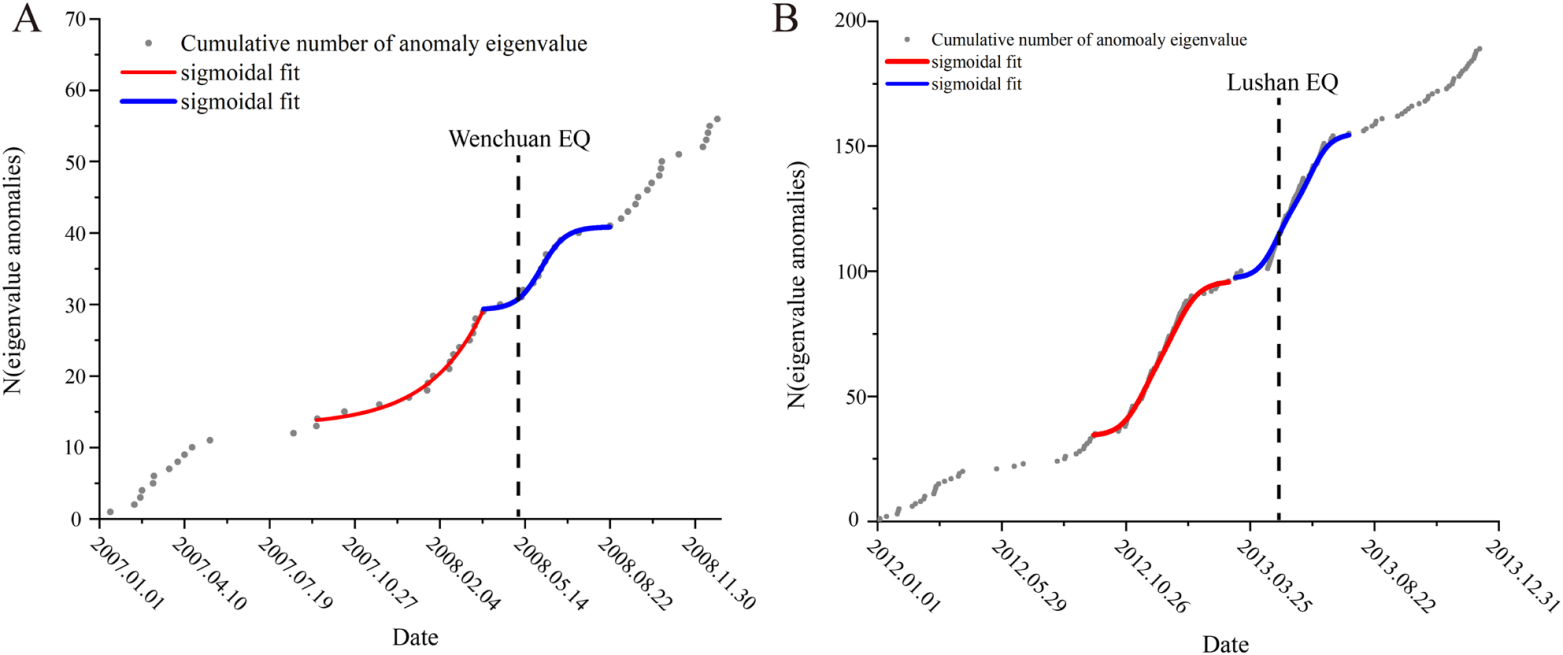
Accumulated results of abnormal days of boreholes strain data in Guza station. (**A**) is the cumulative result of abnormal days during the Wenchuan earthquake, and the red and blue lines represent the results of the sigmoidal fitting; (**B**) is the cumulative result of abnormal days during the Lushan earthquake, and the red and blue lines represent the results of the sigmoidal fitting.

Figure 7 illustrates the temporal behavior of N(t), denoted here as N (number of abnormal day). The day of the earthquake is indicated by a dotted vertical line. The red and blue curves represent the sigmoidal fits for the period near the earthquake occurrence.

The sigmoidal temporal behavior, as shown in Fig. 7A, displayed a two-part concavity: one portion shows a sharp increase in the number of anomalous days starting in September 2007 and the other part begins from March 2008. Reorganization of strains in the crust following an earthquake of this magnitude typically results in a significant number of anomaly events. There was a sharp increase in the number of anomaly days following the earthquake, as seen in Fig. 7A. Chi et al. used a methodology similar to ours to analyze borehole strain data from Guza station and the sigmoidal temporal behavior showed an acceleration about 4 months (starting in January 2008) before the Wenchuan earthquake^20^. Wang et al.^45^ employed a method based on the inversion of regional seismic source mechanisms to quantitatively determine stress tensors for the Wenchuan earthquake. Their analysis indicated that from June 2007 until the main Wenchuan earthquake, there was a notable increase in local stress levels. This suggests a predominant accumulation of stress before reaching its peak, followed by a stable release after the peak. Shi et al.^46^ calculated the pre-seismic b-value changes for the Wenchuan earthquake and observed a continuous declining trend from mid-2007 until the earthquake. The rate of this decline accelerated, with a significant drop in the b-value at the beginning of 2008. These research findings align well with the anomaly periods identified in our study and we extracted similar abnormal change processes and also extracted more abnormal days.

In this study, a strikingly similar phenomenon was observed prior to the Lushan earthquake. As illustrated in Fig. 7B, the sigmoidal temporal pattern also displayed a two-part concavity: the first part exhibited a sharp increase in the number of anomalous days starting from late December 2012, while the second part emerged from early 2013 onwards. Yu et al.^20^ used the approximate negentropy (ApNe) and b value to analyze the Lushan earthquake, their results showed an abrupt acceleration followed by moderate acceleration, which indicates that non-Gaussian changes in the borehole strain data began to appear. Xu et al.^47^ analyzed the time series of GNSS baseline length changes prior to the Lushan earthquake. Their findings revealed abnormal deviations in the trend of GNSS baseline changes between September and December 2012; Qiu et al.^48^ concluded that the abnormal changes observed at the Guza Station (several days before the earthquake) should be related to the Lushan earthquake. Chi et al.^49^ also observed abnormal strain 5 months before the Lushan earthquake that lasted for 3 months. The aforementioned results are consistent with the anomaly periods identified in this study. This also proves the effectiveness of the method in this paper.

**Figure 8:**
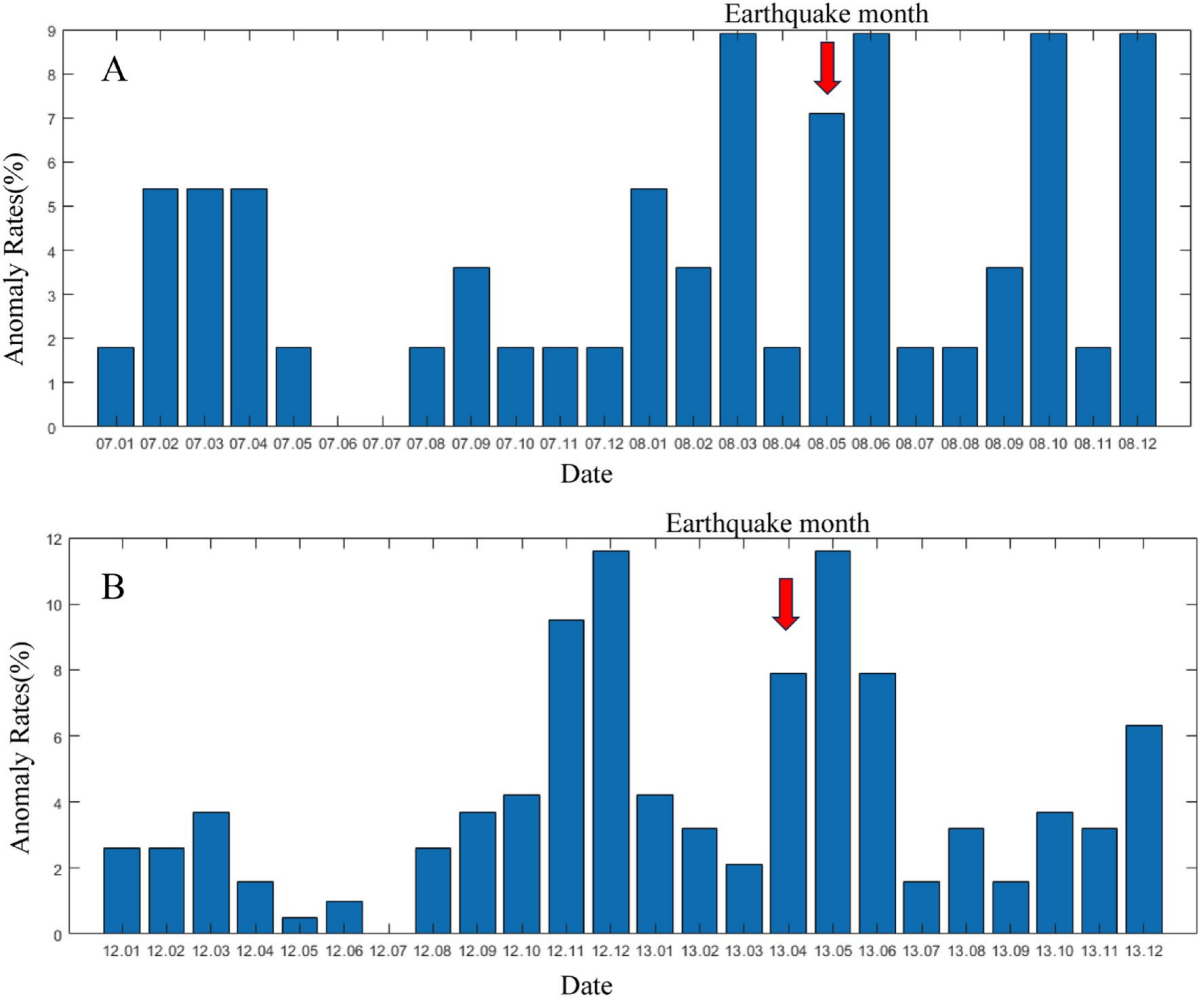
The monthly anomaly rate chart for the two earthquakes. (**A**) illustrates the monthly anomaly rate chart for the Wenchuan earthquake, with the red arrow indicating the month in which the earthquake occurred; (**B**) illustrates the monthly anomaly rate chart for the Lushan earthquake, with the red arrow indicating the month in which the earthquake occurred.

As depicted in Fig. 7B, akin to the abnormal alterations preceding the Wenchuan earthquake, the change of the number of anomalies days prior to the Lushan earthquake also demonstrated two distinct phases of change. Ma et al*.* suggest that interactions between various segments of a fault cause these regions to evolve from acting independently to displaying coordinated behavior. The extent of this coordination in fault activity serves as an indicator of the fault's stress condition. The progression of this coordination typically encompasses a deviation from the linear stage and meta-instability stage^50^. As depicted in Fig. 7, notable anomalies began to manifest 6 months prior to the Wenchuan and Lushan earthquakes. These irregularities align with the deviation from the linear stage, signifying a period of stress accumulation. Furthermore, a surge in anomalies was evident 2–3 months and even just days, before the earthquake, illuminating the stress redistribution that characterizes the fault's meta-instability stage. The main earthquake event mirrors a phase of strain instability and the subsequent wave of anomalies can be attributed to the ensuing aftershocks. Both earthquakes showed a process of fault transition from steady state to instability: during the first stress loading phase (indicated by the red fit curve), the crustal instability threshold was not reached. However, in the course of the second stress loading phase (represented by the blue fit curve), the crust ruptured, ultimately leading to an earthquake. This progression may mirror the entire process from crustal stress loading to instability and it also aligns with the synergistic process of fault interactions^50^. We plotted the anomaly rate chart. As shown in the Fig. 8, A is the monthly anomaly rate chart for the Wenchuan earthquake; B is the monthly anomaly rate chart for the Lushan earthquake, a significant number of anomalies occurred before and after the earthquake, without exhibiting periodic variations.

**Figure 9:**
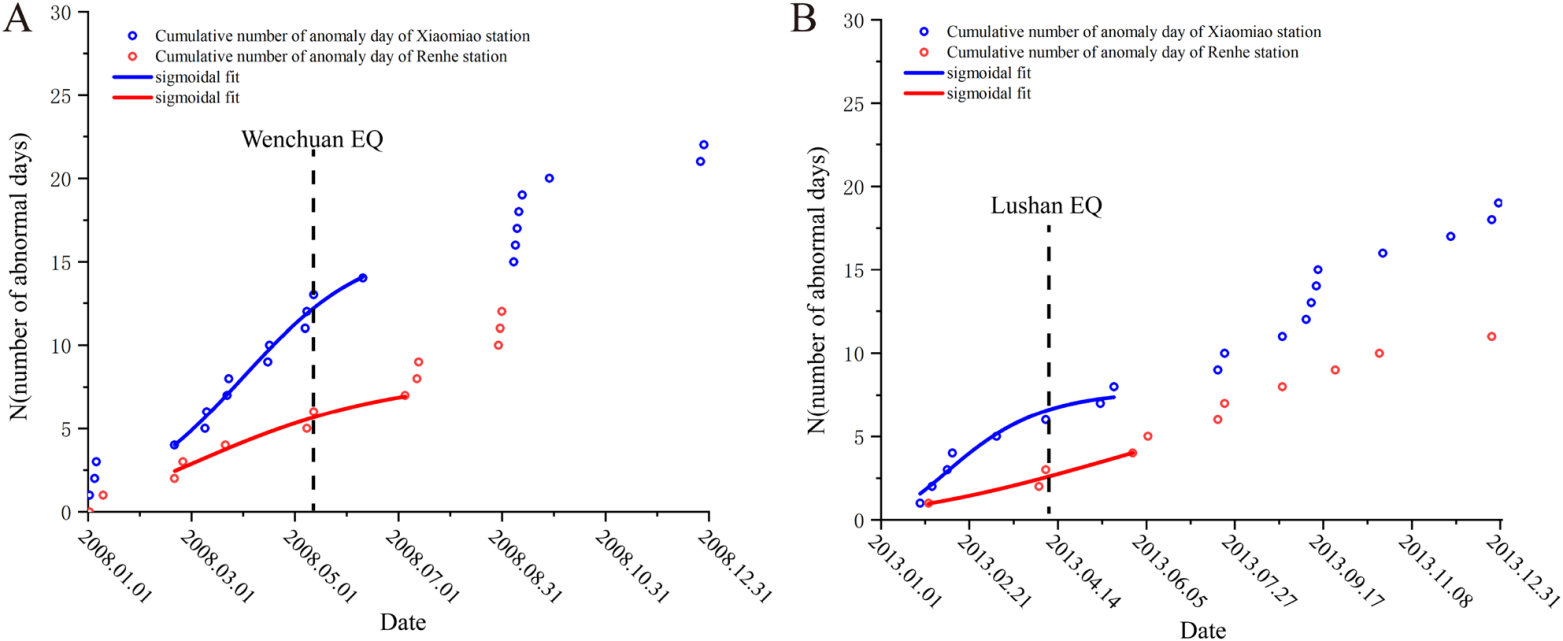
Accumulated results of abnormal days of boreholes strain data in Xiaomiao station and Renhe station. (**A**) is accumulated results of abnormal days of Wenchuan earthquake at two stations, and the red and blue lines represent the results of the sigmoidal fitting. (**B**) is accumulated results of abnormal days of Lushan earthquake at two stations, and the red and blue lines represent the results of the sigmoidal fitting.

The results of our analysis using the same hypothesis for the data from Xiaomiao and Renhe stations for the Wenchuan and Lushan earthquakes are displayed in Fig. 9. Su^51^ conducted calculations and analyses on the earthquake monitoring capability of borehole strainmeters. Through empirical formulas, he deduced that for earthquakes above magnitude 7, the range over which precursory signals of borehole strain observations spread is: 210 km for long-term precursors and 472 km for short-term precursors. The two comparison stations are most likely to detect short-term precursors. Short-term precursors generally refer to anomalies that appear within 3 months of the earthquake. Therefore, this study only selected data from the year of the earthquake at the two stations for comparison.

As is shown in Fig. 9A, the co-seismic signal of the Wenchuan earthquake has been received by both Xiaomiao Station and Renhe Station. By fitting the curve, it can be observed that Renhe Station did not exhibit a similar behavior prior to Wenchuan, whereas Xiaomiao Station started to exhibit an abnormally high daily growth around March and continued to do so until the earthquake took place. According to Fig. 9B’s fitting curve, the Xiaomiao station experienced similar events before the Lushan earthquake as it did before the Wenchuan earthquake. Xiaomiao Station also existed prior to both earthquakes, proving that Guza Station is not the only station experiencing abnormal phenomena. From another perspective, it demonstrates that the anomalies detected by Guza Station are not random occurrences but rather are connected to the two earthquakes. While there are no similar anomalous occurrences at Renhe Station, there are much fewer abnormal days documented at Xiaomiao Station than at Guza Station. The findings of this article are compatible with theory for all of these, which are connected to the distance between the station and the epicenter.

**Figure 10:**
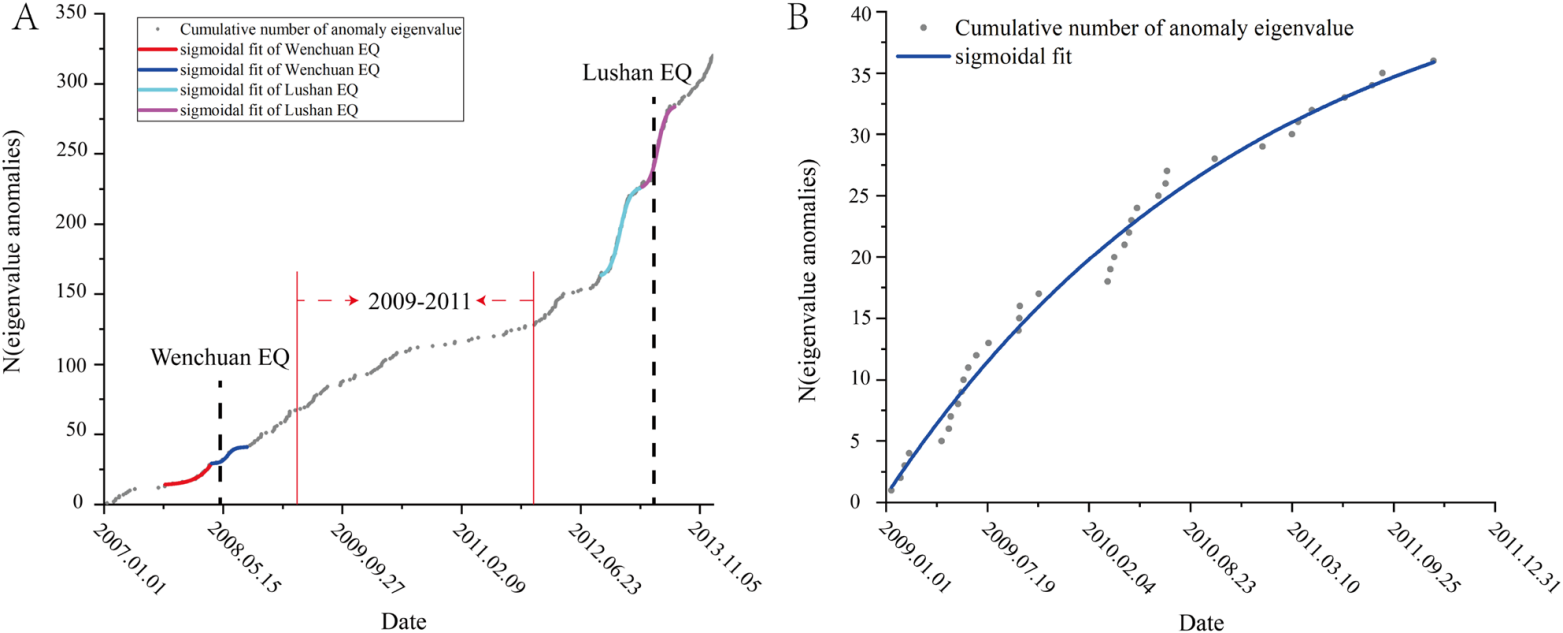
Accumulated results of abnormal days of boreholes strain data in Guza station; (**A**) represents the anomaly extraction results from 2007 to 2013; (**B**) displays the cumulative fitting curve of anomalies from 2009 to 2011.

The Guza station exhibited similar anomaly variations before both earthquakes. To rule out the possibility that this phenomenon occurs periodically, we analyzed the data from 2009 to 2011 using the same network and parameters, as shown in Fig. 10.

As illustrated by Fig. 10B, during the relatively calm period from 2009 to 2011, there are fewer anomalies, and the trend of anomaly changes largely presents as linear. In contrast, in Fig. 10A, around the times of the two earthquakes, there's a higher number of anomalies and the trend exhibits phase-like variations. By comparing anomaly changes across different time frames and aligning them with existing research findings, we have good reason to believe that the anomaly features extracted around the two earthquakes in this study are credible.

The analysis of the anomalies extracted from the Guza station's borehole strain data during the Wenchuan and Lushan earthquakes reveals several key insights. The presence of similar S-shaped rising trends in the cumulative anomaly days preceding both earthquakes suggest a commonality in the pre-earthquake stress accumulation and release processes^52^. Furthermore, the observation of pre-earthquake anomalies at distant stations, such as Xiaomiao and Renhe, supports the validity of the Guza station’s findings. Zhu et al., by comparing the temperature, atmospheric pressure, rainfall and borehole water level data during the two earthquakes, concluded that the anomalies before the two earthquakes were not related to these influencing factors^25,53^.

Several factors could contribute to the observed similarities in pre-earthquake anomalies at the Guza station for the Wenchuan and Lushan earthquakes^54,55^:

1. Shared patterns in crustal stress accumulation and release: The observed pre-earthquake anomalies may reflect a general pattern in the crustal stress accumulation and release process, which could manifest in different earthquakes, leading to similar anomalies observed at the Guza station.
2. Proximity of earthquake epicenters: The relatively close proximity of the Wenchuan and Lushan earthquakes may imply similarities in their causation or seismic mechanisms, potentially resulting in similar pre-earthquake anomalies.
3. Comparable geological settings: The Wenchuan and Lushan earthquakes may share similar geological settings, which could cause the crustal stress accumulation and release processes to exhibit similar characteristics, thus, similar pre-earthquake anomalies.
4. Consistency in monitoring techniques and methodologies: The techniques and methods employed at the Guza station for monitoring the Wenchuan and Lushan earthquakes are consistent, resulting in the extraction of similar anomaly features during data processing and analysis.
5. Coincidence: Although several potential reasons have been identified, it is still possible that the similar anomalies observed at the Guza station before the two earthquakes are coincidental. Further analysis of additional earthquake events and long-term monitoring data is required to validate the generality of this similarity.

From the research presented in this paper, the significance of long-term monitoring becomes evident: The accumulation of stress and strain in the Earth’s crust is a gradual process, and it's only through sustained observation that these subtle changes can be accurately captured. Such changes might be indicative of potential seismic activities. Long-term observation provides continuous underground strain data, crucial for understanding and predicting crustal dynamics, seismic precursors, and the cyclicity of seismic activities. Moreover, extended observation data aids seismologists in refining and improving earthquake prediction models, enhancing their accuracy. However, with these benefits come associated challenges. Equipment maintenance: even though borehole strainmeters are relatively stable, regular maintenance and calibration are imperative to ensure the precision and reliability of the data. Data volume: extended monitoring generates vast amounts of data, necessitating robust storage, processing, and analysis capabilities. Data interpretation: long-term strain data can be influenced by a myriad of complex factors, which elevates the intricacy of its interpretation. In essence, while the prolonged monitoring of borehole strainmeters brings forth invaluable insights, it also introduces its set of challenges that must be adeptly managed.

In conclusion, the similarities in pre-earthquake anomalies at the Guza station for the Wenchuan and Lushan earthquakes may be attributed to shared patterns in crustal stress accumulation and release, proximity of earthquake epicenters, comparable geological settings, consistency in monitoring techniques and methodologies, and coincidence. A more comprehensive understanding of this similarity can be achieved through the analysis of more earthquake events and continuous monitoring of pre-earthquake anomalies in different regions and earthquake types. This will help enhance our understanding of earthquake mechanisms and improve the accuracy of earthquake prediction.

## Conclusion

In this paper, we propose an anomaly detection method based on SVMD and GRU-LUBE. SVMD preserves correlations between data and significantly reduces the time needed for data decomposition. GRU, with its unique structure, can effectively learn and predict data, while LUBE efficiently extracts data anomalies. Statistical analysis of the borehole strain at the Guza station revealed that anomalies prior to the Lushan earthquake were similar to those observed before the Wenchuan earthquake. By comparing the results from Xiaomiao Station and Renhe Station, we demonstrate that the pre-earthquake anomalies extracted by the Guza station are linked to both earthquakes. This suggests that the effective anomaly extraction method employed at the Guza station has successfully identified pre-earthquake anomalies in borehole strain data. The similarity of the cumulative anomaly days graphs for the Wenchuan and Lushan earthquakes, characterized by two S-shaped rising trends, implies the presence of common pre-earthquake characteristics or a shared pattern in crustal stress accumulation and release. This study highlights the importance of further investigation into the underlying mechanisms of pre-earthquake anomalies and the development of advanced techniques for better earthquake prediction and preparedness.

## Data Availability

The data that support the findings of this study are available from the National Institute of Natural Hazards, but restrictions apply to the availability of these data, which were used under license for the current study, so are not publicly available. Data are however available from the first author (Email: 575104711@qq.com) upon reasonable request and with permission of the National Institute of Natural Hazards.
